# Single and multiple phenotype QTL analyses of downy mildew resistance in interspecific grapevines

**DOI:** 10.1007/s00122-018-3065-y

**Published:** 2018-02-07

**Authors:** Konstantin Divilov, Paola Barba, Lance Cadle-Davidson, Bruce I. Reisch

**Affiliations:** 1 Plant Breeding and Genetics Section, School of Integrative Plant Science, Cornell University, Ithaca, NY USA; 2 USDA-ARS Grape Genetics Research Unit, Geneva, NY USA; 3 Horticulture Section, School of Integrative Plant Science, New York State Agricultural Experiment Station, Cornell University, Geneva, NY USA; 4 Instituto de Investigaciones Agropecuarias, INIA La Platina, Santiago, Chile

## Abstract

**Key message:**

Downy mildew resistance across days post-inoculation, experiments, and years in two interspecific grapevine F_1_ families was investigated using linear mixed models and Bayesian networks, and five new QTL were identified.

**Abstract:**

Breeding grapevines for downy mildew disease resistance has traditionally relied on qualitative gene resistance, which can be overcome by pathogen evolution. Analyzing two interspecific F_1_ families, both having ancestry derived from *Vitis vinifera* and wild North American *Vitis* species, across 2 years and multiple experiments, we found multiple loci associated with downy mildew sporulation and hypersensitive response in both families using a single phenotype model. The loci explained between 7 and 17% of the variance for either phenotype, suggesting a complex genetic architecture for these traits in the two families studied. For two loci, we used RNA-Seq to detect differentially transcribed genes and found that the candidate genes at these loci were likely not NBS-LRR genes. Additionally, using a multiple phenotype Bayesian network analysis, we found effects between the leaf trichome density, hypersensitive response, and sporulation phenotypes. Moderate–high heritabilities were found for all three phenotypes, suggesting that selection for downy mildew resistance is an achievable goal by breeding for either physical- or non-physical-based resistance mechanisms, with the combination of the two possibly providing durable resistance.

**Electronic supplementary material:**

The online version of this article (doi:10.1007/s00122-018-3065-y) contains supplementary material, which is available to authorized users.

## Introduction

Downy mildew resistance in grapevine has been mapped to over a dozen loci from over half a dozen *Vitis* spp. (Buonassisi et al. 2017). Most loci found have been qualitative (i.e., they explain the majority of the variation in the disease phenotype in a particular experiment), but a few explain only a small portion of the variation (Bellin et al. 2009; Blasi et al. 2011; Moreira et al. 2011; Venuti et al. 2013). Resistance dependent on a single dominant locus is not seen as being durable, especially to an air-borne outcrossing pathogen like the one that causes grapevine downy mildew (Buonassisi et al. 2017; McDonald and Linde 2002). By contrast, quantitative resistance is controlled by many genes, each of which contributes a small portion to the resistance phenotype (Poland et al. 2009). Additionally, quantitative resistance can be controlled by different metabolic processes in the plant, including nucleotide-binding site leucine-rich repeat (NBS-LRR) resistance genes. A pathogen evolving to overcome quantitative resistance would face a more difficult path to affect the same phenotype, e.g., necrosis or sporulation, as it would on a susceptible plant because more effector genes in the pathogen would need to mutate or experience recombination in order to overcome the multifaceted resistance in the plant. It is especially important to prevent pathogen evolution from overcoming disease resistance in grapevine because it is not economically feasible to replant a vineyard due to loss of disease resistance if it occurs a few years after planting, unlike maize or wheat where one can change the cultivar that is grown yearly.

Grapevine downy mildew, caused by the obligate biotrophic oomycete *Plasmopara viticola* (Berk. & M.A. Curtis) Berl. & de Toni, is a common cause of yield loss for *Vitis vinifera* cultivars, which generally lack genetic resistance to the disease (Buonassisi et al. 2017). Wild *Vitis* species, e.g., *V. amurensis*, *V. cinerea*, *V. riparia*, *V. rupestris*, on the other hand, have genetic resistance to downy mildew (Cadle-Davidson 2008) and are commonly used as parents in resistance breeding efforts and in genetic studies. Infection on grapevine leaves can be detected by observing sporulation or host necrosis, which on resistant vines is often associated with a hypersensitive response (HR) (Bellin et al. 2009; Buonassisi et al. 2017). Sporulation can be quantified either manually using human vision or by computer vision algorithms (Divilov et al. 2017). Research in identifying downy mildew resistance quantitative trait loci (QTL) in grapevine has focused on disease phenotypes, e.g., sporulation and HR, but physical barriers produced by a plant can also play a role in the prevention of disease. Kortekamp and Zyprian (1999) used four wild *Vitis* accessions to demonstrate that trichomes on the abaxial side of grapevine leaves can play a role in resistance to downy mildew disease by forming a hydrophobic surface above the leaf that blocks *P. viticola* sporangia from reaching stomata. As with sporulation, leaf trichome density can also be quantified using computer vision algorithms (Divilov et al. 2017).

To breed grapevines that have either qualitative or quantitative resistance to downy mildew using marker-assisted selection, one needs to find significant associations between downy mildew resistance ratings and genetic markers. However, it is rare to identify potential causal genes after QTL mapping due to linkage disequilibrium present in the region a QTL resides. Therefore, one often knows the physical location of the causal locus only within a range of one to four megabases. RNA-Seq (Wang et al. 2009) is a high-throughput RNA sequencing method that can be used to identify candidate genes for QTL by finding differentially transcribed genes in the region where a QTL resides. Here, we describe newly identified QTL associated with downy mildew resistance phenotypes, and the use of RNA-Seq to find candidate genes for two QTL on chromosome 14.

## Materials and methods

### Plant material and phenotyping methods

The plant material used consisted of two F_1_ grapevine families, *V. rupestris* B38 x ‘Horizon’ (RH) and ‘Horizon’ × *V. cinerea* B9 (HC), grown unreplicated in a vineyard in Geneva, New York. ‘Horizon’ ancestry is derived from *V. vinifera* and North American *Vitis* spp. (Reisch et al. 1983). The progeny of the HC family segregate for ribbon-type trichomes (Ma et al. 2016) on the abaxial side of their leaves, while leaves of the RH family are glabrous. Both families were phenotyped following controlled inoculation of eightfold replicated, surface-sterilized, 1-cm leaf discs on 1% agar, as previously described in detail (Divilov et al. 2017) and briefly summarized here. Phenotypes were assayed with manual and computer vision methods. The manual rating of sporulation area was on an ordinal scale from 1 = no sporulation to 5 = extensive sporulation (Kono et al. 2015). The computer vision ratings of percent sporulation area ranged between 0 and 1. These were based on analyses of images using OpenCV (Bradski 2000) collected on a handheld Apple iPhone 5s with ambient laboratory lighting, focusing on the blue layer and accounting for unequal lighting conditions using a Wallis filter. The proportion of saturated pixels (value = 255) was calculated after removing leaf veins via the Hough line transform algorithm.

**Fig. 1:**
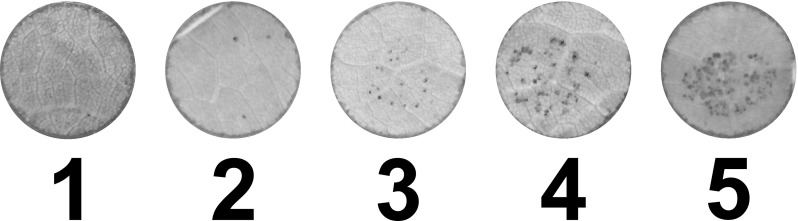
The ordinal visual scale for rating hypersensitive response on grapevine leaf discs

The HC family was also phenotyped for percent leaf trichome area using a leaf disc assay with the same computer vision method at 2 days post-inoculation (dpi) (Divilov et al. 2017). Hypersensitive response (HR) was assessed in both families at 2 dpi using a visual manual rating method where leaf discs were scored on a 1–5 ordinal scale (Fig. 1). The RH family was phenotyped in 2015 with 163 F_1_ genotypes and with three experiments, and in 2016 with 157 F_1_ genotypes and with two experiments. The HC family was phenotyped in 2015 and 2016 with 152 and 145 F_1_ genotypes, respectively, and with two experiments in each year. Susceptible and resistant controls were included to ensure inoculum quality. For the RH family, ‘Cabernet Sauvignon’ was the susceptible control for the first two experiments in 2015, while ‘Chardonnay’ was the susceptible control for all other experiments, and *V. rupestris* B38 was the resistant control. For the HC family, ‘Chardonnay’ was the susceptible control and *V. cinerea* B9 was the resistant control. Each experiment took place at a different date and consisted of four phenotypes taken on successive days starting at either 3 or 4 dpi for the sporulation phenotype. A phenotype within each dpi consisted of the average rating of eight leaf discs, two from each of four leaves obtained from different shoots on a vine. The RH family was phenotyped for HR in all experiments while the HC family was phenotyped for HR in all experiments except the first one in 2015.

### Phenotype modeling and heritability analysis

To analyze the 2015 and 2016 multi-experiment data for a phenotype, we fit a linear mixed model (McCulloch and Searle 2001) ${\bf{y}} = {\bf{Xb}} + {\bf{Z}}_{\text{g}} {\bf{u}}_{\text{g}} + {\bf{Z}}_{\text{gy}} {\bf{u}}_{\text{gy}} + {\boldsymbol \varepsilon}$ for each family individually. ${\bf{X}}$ was a design matrix and ${\bf{b}}$ was a vector of fixed effects. For the HR phenotype, ${\bf{X}}$ contained indicator variables for the intercept and experiments within years. For the manual and computer vision sporulation phenotypes, additional covariates of dpi within experiments within years were included. For the computer vision sporulation phenotype for the HC family, additional covariates of leaf trichomes within experiments within years were included. ${\bf{Z}}_{\text{g}}$ and ${\bf{Z}}_{\text{gy}}$ were incidence matrices relating genotypes to phenotypes and genotypes within years to phenotypes, respectively. ${\bf{u}}_{\text{g}} \sim {\text{N}}({\bf 0}, {\bf{I}}\sigma_{\text{g}}^{2} )$ was a vector of normally distributed genetic effects, also known as estimated breeding values, corresponding to each genotype, where ${\bf{I}}$ is an identity matrix. We used an identity matrix as our genotype covariance matrix because the F_1_ genotypes within each family had no population structure. ${\bf {u}}_{\text{gy}} \sim {\text{N}}({\bf {0}}, {\bf {E}} \otimes {\bf {I}}{\boldsymbol \sigma}_{\text{gy}}^{2} )$ was a vector of normally distributed genotype-by-year interaction effects corresponding to each genotype in a year, where ⨂ is the Kronecker product and ${\bf{E}}$ is an identity matrix with the number of rows and columns equal to the number of years. The genotype-by-year interaction covariance matrix assumes that genotypic performance in 2015 was independent of genotypic performance in 2016. ${\boldsymbol {\varepsilon}} \sim {\text {N}}({\bf {0}},{\bf {I}} {\boldsymbol{\sigma}}_{\varepsilon }^{2})$ was a vector holding the normally distributed independent noise, or error, of the phenotypes. From this model, the broad-sense heritability was estimated as $\frac{{\sigma_{g}^{2} }}{{\sigma_{\text{g}}^{2} + \frac{{\sigma_{\text{gy}}^{2} }}{\text{y}} + \frac{{\sigma_{\varepsilon }^{2} }}{\text{n}}}}$ where $\sigma_{\text{g}}^{2}$, $\sigma_{\text{gy}}^{2}$, $\sigma_{\varepsilon }^{2}$ were the genetic, genotype-by-year interaction, and error variances, respectively, and ${\text{y}}$ and n were the number of years and number of experiments over the years, respectively. In addition to the sporulation and HR phenotypes, we also calculated the heritability of the leaf trichome phenotype. In that case, the only fixed effects in the linear mixed model were the same as those for HR. The models were fit using the EMMREML R package (Akdemir and Godfrey 2015) with the efficient mixed-model association (EMMA) algorithm (Kang et al. 2008) used to obtain estimates of variance components.

### Single phenotype QTL analysis

To find QTL for the breeding values of a trait, a Haley–Knott linear regression model was built using forward and backward stepwise selection with R/qtl (Broman et al. 2003). Haley–Knott regression is an approximation to standard interval mapping (Broman and Sen 2009). Interaction effects were not considered for inclusion in the model. The logarithm of odds (LOD) penalty for each trait was determined by 1000 permutation tests with an alpha value of 0.05. Approximate Bayes credible intervals were calculated for QTL and represented the region in which a QTL resides with probability ≥ 0.95. The genotyping error rate used to calculate conditional genotype probabilities was set to 0.001. RH and HC family genetic maps used for the analyses were made with HetMappS and were previously published (Hyma et al. 2015). Imputation of missing single nucleotide polymorphism (SNP) data was performed using the expectation–maximization algorithm in rrBLUP (Endelman 2011). Physical locations of the SNPs in these maps were obtained using the 12X.2 version (URGI 2014) of the grapevine reference genome PN40024 (Jaillon et al. 2007). For comparison purposes, we performed the same stepwise regression analysis on manual and computer vision sporulation and HR phenotypes within years within experiments within dpi to observe what QTL were found using these data.

### Multiple phenotype Bayesian network analysis

To detect effects between multiple phenotypes, as well as the effect of SNPs on phenotypes, we constructed an averaged Bayesian network for each family using the bnlearn R package (Scutari 2010). A similar analysis has been previously done in wheat (Scutari et al. 2014). For the RH family, we constructed a Bayesian network using the manual sporulation and HR breeding values, as well as the SNPs in that family. For the HC family, the leaf trichome breeding values were included as well. We restricted the manual sporulation trait from affecting the HR and leaf trichome traits, and we restricted the HR trait from affecting the leaf trichome trait. This was done because necrosis is present on the leaf prior to the appearance of sporulation, and leaf trichomes are present on the leaf prior to inoculation. All traits were restricted from affecting the SNPs. We used the SI-HITON-PC algorithm (Aliferis et al. 2010) to find the Markov blanket of each trait individually. The Markov blanket (Pearl 1988) represents the set of traits and SNPs such that a given trait, conditional on its Markov blanket, is independent of all other traits and SNPs. Independence was determined by non-significance of the Pearson’s correlation coefficient between a variable conditional on its Markov blanket and a variable outside the Markov blanket tested using a Student’s *t* test with an alpha value of 0.01. The hill-climbing algorithm (Scutari 2010) was then used to find the structure of the Bayesian network containing the traits and their Markov blankets. In the network, the distribution of a variable, or node, of interest conditional on its parents, i.e., nodes with arrows pointing to the node of interest, is parameterized as a linear regression model. The hill-climbing algorithm greedily adds arrows between nodes such that the total Bayesian information criterion (BIC), which is a function of the log likelihood of a linear regression model and a parameter penalization term, among the linear regression models achieves its highest value (the BIC is rescaled by − 2 in bnlearn). The total BIC is the sum of the BICs of the individual linear regression models. Hill-climbing algorithms can also remove and reverse arrows between nodes, but this was not done here. We allowed the SNPs within all Markov blankets to affect any trait to account for possible pleiotropy. We constructed 1000 networks using a random set of 90% of the individuals in a family for each network and then created an averaged network where we kept the structural components, i.e., the arrows or edges, present in at least half of the networks. Empirically, we found that edge thresholds estimated from data (Scutari and Nagarajan 2013), which were close to 0.5, did not lead to robust networks, i.e., network nodes were different in various iterations of the creation of the averaged network. However, this was not the case for a threshold set to 0.5. QTL bootstrapped confidence intervals of the averaged network SNPs were obtained by calculating the range of physical locations of SNPs on the same chromosome as the averaged network SNPs found to affect the same trait in at least 5% of the networks. The networks were drawn using BayesNet (Luttinen 2013).

### RNA-Seq experimental design and analysis

On 11 July 2016 and 19 July 2016, two leaf disc assay experiments were conducted following the same phenotyping methodology used in the experiments described above. For RNA-Seq analyses, 28 RH genotypes were chosen that segregated for two QTL on chromosome 14, one on each of the RH parental maps found using the stepwise regression approach explained above with the computer vision sporulation trait. Specifically, 15 of the 28 genotypes were heterozygous and homozygous for the most significant marker within the QTL from *V. rupestris* B38 and ‘Horizon’, respectively, while the other 13 genotypes were homozygous and heterozygous for the most significant marker within the QTL from *V. rupestris* B38 and ‘Horizon’, respectively. Because markers exist in very close linkage to the most significant markers in both QTL credible intervals that are of opposite phase, the phase information of the most significant marker in both QTL credible intervals is not informative. Positive and negative control genotypes were included to ensure inoculum quality. At 7 h post-inoculation, single leaf discs from each of the 28 genotypes were frozen in liquid nitrogen and stored at − 80 °C prior to RNA extraction using the Spectrum™ Plant Total RNA Kit (Sigma-Aldrich). Leaf discs of each genotype from the two experiments were combined prior to RNA extraction. Libraries were made using the protocol of Zhong et al. (2011) and sequenced using the Illumina NextSeq 500 to obtain single-end 75 bp reads. Reads were aligned to the 12X.2 version of the grapevine reference genome PN40024 using HISAT2 (Kim et al. 2015) and the transcriptome was assembled using StringTie (Pertea et al. 2015) with the CRIBI functional annotation (Vitulo et al. 2014). Transcribed genes were analyzed by fitting a linear model ${\bf{y}} = {\bf{Xb}} + {\boldsymbol \varepsilon}$ using Ballgown (Frazee et al. 2015) where ${\bf {y}}$ was a vector holding the log_2_(FPKM + 1) values of genes with FPKM (Fragments Per Kilobase of transcript per Million reads sequenced) variances greater than one for each genotype; ${\bf {X}}$ was a design matrix that contained the indicator variables for the intercept and the genotype-phase grouping; ${\bf {b}}$ was a vector holding the mean and the effect of the group value; and ${\boldsymbol {\varepsilon}} \sim {\text {N}}({\bf {0}},{\bf {I}}{\boldsymbol{\sigma}}_{\boldsymbol {\varepsilon}}^{2} )$ was a vector holding the error. For each gene, the full model was compared to a model without the grouping covariate to derive an F statistic. The significance threshold for the F statistic was set to a q value of 0.05. We called genes that passed the significance threshold differentially transcribed genes. Only those physically located within the 95% approximate Bayes credible intervals of the two QTL on chromosome 14 from *V. rupestris* B38 and ‘Horizon’ were considered as possible candidate genes. The UniProt database (The UniProt Consortium 2017) was used to determine the names and GO terms of candidate genes.

### Data availability

The phenotypic and genetic data, as well as the code used to run the linear mixed models and single time point, single phenotype, and multiple phenotype Bayesian network analyses, are available at https://github.com/kdivilov/TAG_2018. The RNA-Seq analysis pipeline is included in the repository as well. The raw RNA-Seq data are available from https://www.ncbi.nlm.nih.gov/bioproject/PRJNA281110.

## Results

All phenotypes were approximately normally distributed, except for the HC family HR and manual sporulation phenotype distributions, which were approximately truncated normal distributions (Supplementary Fig. 1; Divilov et al. 2017). The heritabilities for the RH family manual and computer vision sporulation and HR phenotypes were 0.40, 0.43, and 0.58, respectively. The heritabilities for the HC family manual and computer vision sporulation and HR phenotypes were 0.67, 0.21, and 0.73, respectively. The heritability of leaf trichomes in the HC family was 0.83. Single phenotype and multiple phenotype Bayesian network analyses in total identified ten significant QTL for these traits on chromosomes 5, 6, 7, 8, 11, 14 (two QTL), 15, 16, and 18, described below.

Single phenotype QTL from the RH family explained between 7 and 17% of the variation in the traits examined. Three QTL, one on chromosome 11 from ‘Horizon’ and two on chromosome 14 from *V. rupestris* B38 and ‘Horizon’ were found using the computer vision sporulation breeding values (Table 1). The same QTL with overlapping physical locations were found using the manual sporulation breeding values in addition to one on chromosome 18 from ‘Horizon’. Two QTL, one on chromosome 8 from ‘Horizon’ and one on chromosome 11 from *V. rupestris* B38 and ‘Horizon’ were found using the HR breeding values. The HR QTL on chromosome 11 co-located with the sporulation QTL on chromosome 11.

**Table 1:** Statistics for the QTL found from the single phenotype stepwise regression analysis performed on breeding values of the *V. rupestris* B38 x ‘Horizon’ (RH) and ‘Horizon’ × *V. cinerea* B9 (HC) F_1_ families incorporating data across 2 years and multiple experiments

Name	Family	Chr	Heterozygous parent^a^	Phenotype	LOD threshold^b^	LOD score^c^	% Var. explained^d^	Effect size^e^	95% credible interval (Mbp)^f^
*Rpv17*	RH	8	Horizon	Hypersensitive response (HR)	3.60	6.70	12.94	0.149	11.369–11.721–12.184
*Rpv18*	RH	11	Horizon	Sporulation (manual)	3.59	4.69	8.77	0.159	7.038–8.138–19.921
*Rpv18*	RH	11	Horizon	Sporulation (computer vision)	3.65	4.21	8.51	0.006	7.038–16.995–19.921
	RH	11	*V. rupestris* B38	Hypersensitive response (HR)	3.60	4.11	7.66	0.117	7.609–17.753–19.857
*Rpv18*	RH	11	Horizon	Hypersensitive response (HR)	3.60	8.71	17.33	0.169	15.397–15.397–16.994
	RH	14	Horizon	Sporulation (manual)	3.59	7.75	15.14	0.208	23.275–24.823–25.002
	RH	14	Horizon	Sporulation (computer vision)	3.65	5.76	11.91	0.007	24.366–25.002–25.778
*Rpv19*	RH	14	*V. rupestris* B38	Sporulation (manual)	3.59	6.19	11.83	0.184	27.119–29.790–29.790
*Rpv19*	RH	14	*V. rupestris* B38	Sporulation (computer vision)	3.65	7.34	15.51	0.008	27.085–29.543–29.790
	RH	18	Horizon	Sporulation (manual)	3.59	3.79	6.99	0.146	6.598–9.684–14.528
	HC	5	Horizon	Sporulation (manual)	3.43	5.58	11.27	0.314	0.844–3.112–5.511
	HC	5	Horizon	Hypersensitive response (HR)	3.44	6.91	15.10	0.177	0.114–2.388–3.642
*Rpv20*	HC	6	Horizon	Hypersensitive response (HR)	3.44	4.00	8.37	0.142	0.770–0.907–13.625
*Rpv21*	HC	7	Horizon	Sporulation (manual)	3.43	5.42	10.90	0.312	0.994–2.129–3.150
	HC	8	Horizon	Sporulation (manual)	3.43	5.81	11.77	0.323	16.814–19.217–22.458
	HC	8	Horizon	Hypersensitive response (HR)	3.44	4.86	10.29	0.147	17.766–19.609–22.458

^a^The heterozygous parent is the one that has the heterozygous allele. SNPs for the genotypes in the families either were homozygous for one allele or heterozygous because only pseudo-testcross markers were used to build the genetic maps (Hyma et al. 2015)

^b^Calculated using 1000 permutation tests with an alpha value of 0.05

^c^Given for the most significant marker in a QTL credible interval

^d^Calculated as $\frac{\text{Type III SS}}{\text{Total SS}} \times 100$ for the most significant marker in a QTL credible interval. Type III sum of squares (SS) of a QTL is the SS of that QTL conditional on all other QTL in the model

^e^The absolute value of the effect of the most significant marker in a QTL credible interval when the other QTL listed for a particular phenotype within a family are included in the model. The absolute value is given because phase information of the most significant marker is not informative due to linkage with markers of opposite phase. The dimension of the effect size is the same as the dimension of the phenotype score

^f^Location intervals are based on the 12X.2 version (URGI 2014) of the grapevine reference genome. The middle value represents the location of the most significant marker

Single phenotype QTL from the HC family explained between 8 and 15% of the variation in the traits examined. Three QTL on chromosomes 5, 7, and 8 from ‘Horizon’ were found using the HC family manual sporulation breeding values, but no QTL were found using the computer vision sporulation breeding values. Three HR QTL from ‘Horizon’ were identified, one each on chromosomes 5 and 8 that co-located with those found with the HC manual sporulation breeding values, and one on chromosome 6. The QTL found on chromosome 8 from the HC family manual sporulation and HR breeding values did not co-locate with the one related to HR in the RH family.

Comparing QTL found using the breeding values derived from the linear mixed model that utilized all the data available to those found using phenotypes from a single dpi within an experiment within a year, fourteen and eight QTL were found with the latter data for RH and HC families, respectively (Supplementary Table 1). Among those, seven and four of the QTL in their respective families were found only once. All five and four QTL found in the RH and HC families, respectively, using the linear mixed model were also found using the individual time point analysis. No QTL that was found only once using the individual time point analysis was found using the linear mixed model approach. For the RH family, the mean and median approximate Bayes credible intervals of QTL obtained using the breeding values were 5.7 and 2.7 Mbp wide, while those obtained using the individual phenotypes were 6.3 and 4.5 Mbp wide, respectively. For the HC family, the mean and median approximate Bayes credible intervals of QTL obtained using the breeding values were 5.6 and 4.7 Mbp, while those obtained using the individual phenotypes were 7.9 and 6.3 Mbp, respectively.

**Fig. 2:**
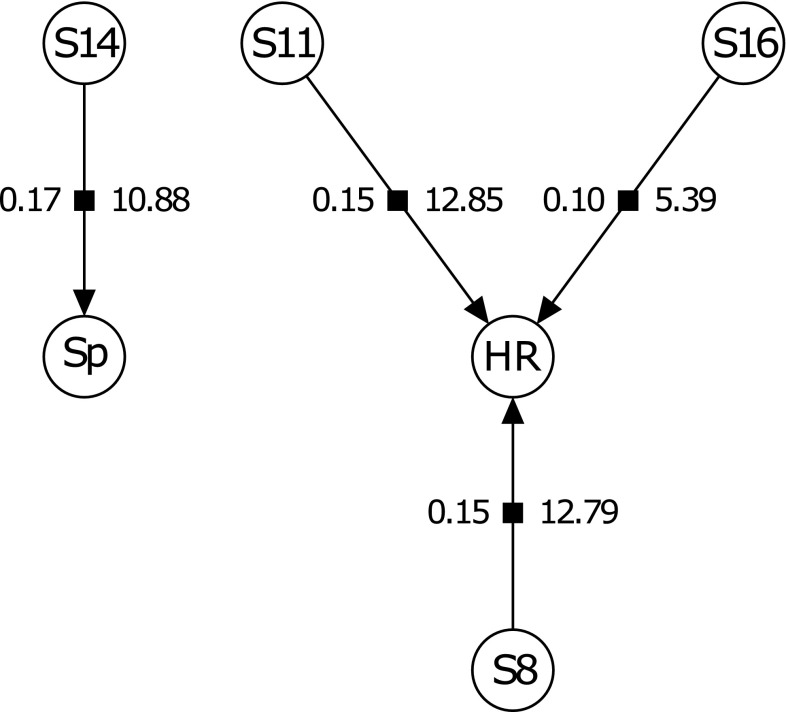
The averaged Bayesian network for the RH family manual sporulation (Sp) and hypersensitive response (HR) traits. S8, S11, S14, and S16 correspond to SNPs on chromosomes 8, 11, 14, and 16, respectively. The number to the left of an edge pointing from a SNP to a trait represents the absolute effect size of the SNP on the trait while the number to the right represents the percent variance of the trait explained by the SNP calculated as $\frac{\text{Type III SS}}{\text{Total SS}}\, \times \,100$. Type III sum of squares (SS) of a SNP is the SS of that SNP conditional on all SNPs in the model. The SNP confidence intervals are given in Table 2

**Fig. 3:**
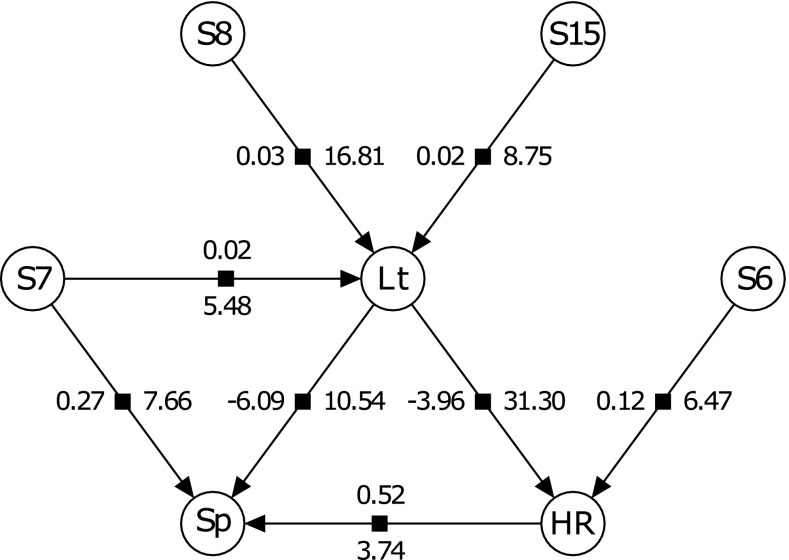
The averaged Bayesian network for the HC family manual sporulation (Sp), hypersensitive response (HR), and leaf trichome (Lt) traits. S6, S7, S8, and S15 correspond to SNPs on chromosomes 6, 7, 8, and 15, respectively. The numbers above and to the left of an edge pointing from a trait/SNP to a trait represents the effect size of the trait/SNP on the trait while the numbers below and to the right represents the percent variance of the trait explained by the trait/SNP calculated as $\frac{\text{Type III SS}}{\text{Total SS}}\, \times \,100$. Type III sum of squares (SS) of a trait/SNP is the SS of that trait/SNP conditional on all other traits and SNPs in the model. Effect sizes from SNPs are absolute values while those from traits are not. The SNP confidence intervals are given in Table 2

The averaged Bayesian network for the RH family showed no effect on sporulation by HR (Fig. 2). A QTL was found to affect sporulation on chromosome 14 from *V. rupestris* B38 that co-located with the one from the single phenotype analysis (Table 2). Three QTL were found to affect HR on chromosomes 8 and 11 from ‘Horizon’ that co-located with those from the single phenotype analysis and on chromosome 16 from *V. rupestris* B38 that was not found using the single phenotype analysis. The averaged Bayesian network for the HC family showed leaf trichomes having a negative effect on both sporulation and HR and HR having a positive effect on sporulation (Fig. 3). A QTL on chromosome 6 from ‘Horizon’ was found to have an effect on HR and a QTL on chromosome 7 from ‘Horizon’ was found to have an effect on both sporulation and leaf trichomes. Two additional QTL on chromosomes 8 and 15 from ‘Horizon’ had an effect on leaf trichomes. The QTL on chromosomes 6, 7, and 8 co-located with those from the single phenotype analysis.

**Table 2:** The physical locations of QTL found using the multiple phenotype Bayesian network analysis performed on breeding values of the *V. rupestris* B38 × ‘Horizon’ (RH) and ‘Horizon’ × *V. cinerea* B9 (HC) F_1_ families incorporating data across 2 years and multiple experiments

Name	Family	Chr	Heterozygous parent^a^	Phenotype(s)	95% confidence interval (Mbp)^b^
*Rpv17*	RH	8	Horizon	Hypersensitive response (HR)	11.656–11.656–11.961
*Rpv18*	RH	11	Horizon	Hypersensitive response (HR)	15.397
*Rpv19*	RH	14	*V. rupestris* B38	Manual sporulation (Sp)	29.543
	RH	16	*V. rupestris* B38	Hypersensitive response (HR)	22.124
*Rpv20*	HC	6	Horizon	Hypersensitive response (HR)	6.64
*Rpv21*	HC	7	Horizon	Manual sporulation (Sp) and leaf trichomes (Lt)	1.455–2.610–4.080
	HC	8	Horizon	Leaf trichomes (Lt)	17.545–17.766–21.504
	HC	15	Horizon	Leaf trichomes (Lt)	17.663

^a^The heterozygous parent is the one that has the heterozygous allele. SNPs for the genotypes in the families either were homozygous for one allele or heterozygous because only pseudo-testcross markers were used to build the genetic maps (Hyma et al. 2015)

^b^Location intervals are based on the 12X.2 version (URGI 2014) of the grapevine reference genome. The middle value represents the location of the markers in the networks in Figs. 2 and 3. The lack of confidence intervals for five out of the eight QTL was due to the absence of other SNPs affecting the phenotype in at least 5% of the networks

Of the RNA-Seq reads from the 28 genotypes, on average 86.5% aligned to the 12X.2 reference genome, ranging from 84.3 to 88.3%. In total, 30,402 genetic loci were detected in the transcriptome, 45% of which had a FPKM variance across the genotypes of less than one. There were 95 differentially transcribed genes, of which 75 were located on chromosome 14. Twenty-seven were within the 95% credible intervals for the two QTL on chromosome 14 from *V. rupestris* B38 and ‘Horizon’. Among these, two did not correspond to those existing in the CRIBI annotation. The 25 remaining genes are listed in Table 3 and their protein GO terms are given in Supplementary Table 2. Nine of the 25 genes were in the credible interval of the QTL from *V. rupestris* B38, while the other 16 were in the credible interval of the QTL from ‘Horizon’.

**Table 3:** A list of differentially transcribed candidate genes within two QTL credible intervals on chromosome 14 from the *V. rupestris* B38 × ‘Horizon’ F_1_ family ordered by significance

Gene ID	UniProt ID	Protein name	Heterozygous parent	Fold change^a^	*q* Value	Transcript range (bp)^b^
VIT_14s0108g01130	F6H5V6	Putative uncharacterized protein	*V. rupestris* B38	0.33|2.97	1 × 10^−11^	29,767,850–29,771,739
VIT_14s0068g00980	D7SVG4	Putative uncharacterized protein	Horizon	1.50|0.67	0.000004	24,740,077–24,753,861
VIT_14s0068g01970	F6H3X5	Putative uncharacterized protein	Horizon	0.57|1.76	0.000005	25,633,234–25,634,255
VIT_14s0108g00040	D7SX63	FACT complex subunit SSRP1	*V. rupestris* B38	0.85|1.17	0.000044	28,871,463–28,878,208
VIT_14s0066g02550	D7TX08	Plasma membrane ATPase	*V. rupestris* B38	1.50|0.66	0.000229	28,745,606–28,754,974
VIT_14s0068g01730	D7SVM8	Putative uncharacterized protein	Horizon	1.51|0.66	0.000245	25,424,955–25,439,887
VIT_14s0068g00800	D7SVE8	Putative uncharacterized protein	Horizon	0.81|1.23	0.000316	24,574,253–24,580,946
VIT_14s0108g00150	F6H5P3	Putative uncharacterized protein	*V. rupestris* B38	1.51|0.66	0.000628	28,944,177–28,951,278
VIT_14s0068g01720	F6H465	Putative uncharacterized protein	Horizon	0.65|1.53	0.002021	25,424,822–25,425,652
VIT_14s0068g01100	F6H429	Putative uncharacterized protein	Horizon	1.19|0.84	0.002182	24,882,721-24,885,537
VIT_14s0068g02110	A5BE40	Putative uncharacterized protein	Horizon	1.43|0.70	0.002228	25,707,772–25,712,673
VIT_14s0068g01940	A5BCW2	Putative uncharacterized protein	Horizon	0.81|1.24	0.002607	25,605,002–25,609,466
VIT_14s0066g02560	A5BD80	Thioredoxin h4	*V. rupestris* B38	0.83|1.20	0.008721	28,755,934–28,756,776
VIT_14s0068g00660	Q19N38	WD repeat 2	Horizon	1.30|0.77	0.010981	24,473,597–24,477,950
VIT_14s0108g01100	F6H5V3	Putative uncharacterized protein	*V. rupestris* B38	1.37|0.73	0.012521	29,753,062–29,756,587
VIT_14s0108g00070	D7SX65	Putative uncharacterized protein	*V. rupestris* B38	0.71|1.40	0.013738	28,889,440–28,894,772
VIT_14s0066g00830	D7TWK7	Putative uncharacterized protein	*V. rupestris* B38	0.75|1.33	0.017500	27,308,643–27,313,582
VIT_14s0068g01310	F6H442	Putative uncharacterized protein	Horizon	0.82|1.22	0.021602	25,043,826–25,044,557
VIT_14s0068g01710	D7SVM6	Putative uncharacterized protein	Horizon	0.75|1.33	0.021602	25,408,934–25,417,805
VIT_14s0066g02380	A5AEQ 0	Putative uncharacterized protein	*V. rupestris* B38	1.16|0.86	0.022765	28,580,980–28,583,873
VIT_14s0068g01840	A5BFT4	Putative uncharacterized protein	Horizon	0.72|1.39	0.024415	25,554,099–25,554,809
VIT_14s0068g01260	F6H440	Putative uncharacterized protein	Horizon	0.58|1.71	0.027264	25,012,954–25,022,155
VIT_14s0068g01990	D7SVP7	Putative uncharacterized protein	Horizon	0.72|1.39	0.028077	25,641,499–25,645,266
VIT_14s0068g01270	F6H441	Putative uncharacterized protein	Horizon	1.84|0.54	0.029598	25,022,156–25,022,479
VIT_14s0068g01160	D7SVI1	Putative uncharacterized protein	Horizon	2.20|0.45	0.048404	24,943,888–24,944,747

GO terms for these genes are given in Supplementary Table 2

^a^Calculated as two raised to a power equal to that of the effect size of the grouping covariate for a given gene. Two fold change values are given due to the uncertainty of which phase the effect is coming from for a given QTL. Each column left or right of the vertical bar represents one phase

^b^The range was calculated by finding the start and end locations of all transcripts for a gene and finding the most proximal start location and the most distal end location

## Discussion

When searching for QTL to introgress into grapevine germplasm, we are particularly interested in those QTL that show a consistent effect across years. In our linear mixed model approach, we were able to take into account the effects of year and genotype-by-year interaction because we had 2 years of phenotypic data. However, the model was naïve with respect to the assumption of independence between years and did not take into account genotype-by-location interaction effects because the F_1_ families were grown in only one location. In the future, environmental variables, such as soil moisture, could be explored to account for genotype-by-environment interactions. This may aid in understanding how QTL perform in different years and locations using only the data from a single location because such environmental variables are not specific to locations. While we replicated within individual time points since each phenotype was the average of eight leaf disc ratings, this replication did not take into account the effect of the environmental conditions prior to leaf harvest. Thus, when analyzing experiments individually for QTL, as we did using the individual time point analysis, there is a greater level of uncertainty surrounding the phenotypes, which play the role of breeding values in the individual time point analysis, than if the genotypes were replicated across multiple experiments. As we replicate genotypes, we are more certain of their estimated breeding values, and, consequently, of the estimated marker effects. This may explain why none of the QTL found only once using the individual time point analysis were found using the single phenotype analysis and why the QTL found using the individual time point phenotypes have much larger credible intervals.

The QTL found in the RH family using the single phenotype analysis were the same for the manual or computer vision sporulation breeding values with the exception of one additional QTL for the manual sporulation breeding values (Table 1). This is consistent with the finding that the two phenotypes are highly correlated (Divilov et al. 2017). The failure to find any QTL using the computer vision sporulation breeding values in the HC family and the low heritability found for that phenotype reflects the poor accounting of lighting conditions in the computer vision system. Image capture with improved lighting could significantly improve the results for families with trichomes. In the absence of trichome segregation in the RH population, downy mildew sporulation has moderate–high heritability. The QTL on chromosomes 5 and 8 from ‘Horizon’ found previously for leaf trichome density using the 2 dpi computer vision rating (Divilov et al. 2017) were found to be QTL for disease resistance when using the manual sporulation and HR breeding values in the single phenotype analysis. This is consistent with our finding that leaf trichomes have an effect on manual sporulation and HR using the multiple phenotype Bayesian network approach. The averaged Bayesian network suggests that the QTL on chromosome 8 operates through the modulation of leaf trichomes (Fig. 3). In the same network, we found that the presence of necrosis (here, loosely termed HR) is associated with an increase in sporulation. While HR is a component of a resistance reaction, it does not always completely stop pathogen colonization, and we did not make direct observations to determine whether HR was associated with infection sites or was spontaneous lesions. Sporulation and HR were not associated in the RH family, and we could not find a similar positive correlation in the literature. Further experimentation would be required to determine the cause of this discrepancy. Likewise, the suggested pleiotropic effect of the QTL on chromosome 7 on sporulation and leaf trichomes would be another interesting mechanism to dissect. The advantage of building a multiple phenotype Bayesian network over a single phenotype QTL search is that one obtains a better understanding of the mechanisms underlying traits. For example, in the single phenotype QTL analyses, we found that QTL on chromosomes 5 and 8 are associated with both sporulation and HR. These same QTL were found to be associated with leaf trichome density (Divilov et al. 2017). The Bayesian network attempts to deconvolute these associations using all the phenotypes, with the resulting network showing that neither of the QTL are pleiotropic as the single phenotype analyses suggested (unlike the QTL on chromosome 7).

While most QTL found using the single phenotype analysis were found using the Bayesian network analysis, some of the QTL did not have confidence intervals in the latter analysis (Table 2). For the RH family, the SNP found in the averaged network to affect a trait was on a different parental map than the other SNPs on the same chromosome found to affect the trait in at least 5% of the networks. For the HC family, only one SNP that was found in the averaged network was associated with the QTL on chromosomes 6 and 15 in at least 5% of the networks. This does not mean that the QTL location is known with certainty, but rather is a result of the robustness of the Markov blanket found for the traits. To obtain a more informative level of uncertainty of the QTL physical positions in such situations, one can obtain an approximate Bayesian credible interval as was done in the single phenotype approach.

There are currently 16 known QTL reported to be associated with downy mildew disease resistance (VIVC 2017). No QTL have been found on chromosomes 6, 8, 11, or 16, where we found QTL associated with sporulation and HR. While QTL on chromosome 14 have previously been found, the QTL found by Blasi et al. (2011) and Venuti et al. (2013) do not physically co-locate to the QTL we found on chromosome 14. The QTL on chromosome 7 found using the HC family single phenotype and Bayesian network analyses did not co-locate to one previously found on chromosome 7 by Moreira et al. (2011). We have designated the QTL on chromosome 8 from ‘Horizon’ *Rpv17*; the QTL on chromosome 11 from ‘Horizon’ *Rpv18*; the QTL on chromosome 14 from *V. rupestris* B38 *Rpv19*; the QTL on chromosome 6 from ‘Horizon’ *Rpv20*; and the QTL on chromosome 7 from ‘Horizon’ *Rpv21*. Because the stepwise regression and Bayesian network analyses disagreed on whether there are one or two QTL on chromosome 14, we only assigned a name to one QTL on that chromosome. Additionally, we have not proposed naming any QTL found only using stepwise regression or only using the Bayesian network analysis.

Since the QTL on chromosome 14 did not explain a large portion of the phenotypic variance, we did not expect to find differentially transcribed NBS-encoding genes, which tend to play a qualitative role in disease resistance, although this role is not absolute (Poland et al. 2009). While a gene encoding FACT (FAcilitates Chromatin Transcription) complex subunit SSRP1 (Structure Specific Recognition Protein 1), which has a GO term associated with DNA binding, was differentially transcribed, we could not find any literature showing its role in disease resistance. Thioredoxin h4, which is part of a family of proteins that interacts with peroxidases (Arnér and Holmgren 2000), is a promising candidate protein because there is evidence that peroxidase activity is associated with downy mildew disease resistance (Kortekamp et al. 1998). The protein A5BE40 likely plays a related role as it is localizes to the peroxisome. Another promising candidate gene is VIT_14s0068g00800, whose protein is predicted to interact in a SNARE (SNAP [Soluble NSF [N-ethylmaleimide-Sensitive Factor] Attachment Protein] REceptor) complex. Proteins in *Arabidopsis thaliana* interacting in SNARE complexes were shown to be responsible for non-host resistance to powdery mildew of barley (Collins et al. 2003). VIT_14s0068g01970, which is involved in xylan biosynthesis, is another promising candidate gene because the presence of xylan, a type of hemicellulose, was shown to be associated with increased infection of *Fusarium herbarum* on wheat (Wingard 1941). Unfortunately, most differentially transcribed genes were associated with proteins of unknown functionality.

In this study using a multi-year, multi-experiment analysis, we have shown that disease resistance in the genotypes studied is a trait controlled by many QTL with small effect sizes, and is influenced by leaf trichomes as well. Five QTL not previously described were identified. For one F_1_ family studied, we have some evidence that the underlying candidate genes are likely not NBS-encoding genes. We used a susceptible *V. vinifera* genome sequence for our analysis as no resistant wild *Vitis* genome has been assembled, so our gene search was partially biased. We believe breeding with these quantitative genes will help to generate durably resistant grapevine cultivars compared to breeding solely with R genes (Poland et al. 2009). Because we found that leaf trichomes have an effect on disease resistance in one F_1_ family, breeding for leaf trichomes presents another opportunity to select for downy mildew disease resistance. We expect that stacking QTL for leaf trichomes and sporulation and HR disease resistance would produce more durable resistance than breeding for one mechanism of resistance alone.

### **Author contribution statement**

KD, LCD, BIR conceived of and designed the experiment. PB made the genetic maps and helped with QTL mapping. KD conducted the phenotyping and statistical analysis and wrote the manuscript.

## Electronic supplementary material

Below is the link to the electronic supplementary material.

Electronic supplementary material 1 (PDF 123 kb)

Electronic supplementary material 2 (XLSX 24 kb)

Electronic supplementary material 3 (XLSX 15 kb)

## References

[CR1] Akdemir D, Godfrey OU (2015) EMMREML: fitting mixed models with known covariance structures. R package version 3.1. https://CRAN.R-project.org/package=EMMREML

[CR2] Aliferis CF, Statnikov A, Tsamardinos I, Mani S, Koutsoukos XD (2010). Local causal and markov blanket induction for causal discovery and feature selection for classification part I: algorithms and empirical evaluation. J Mach Learn Res.

[CR3] Arnér ESJ, Holmgren A (2000). Physiological functions of thioredoxin and thioredoxin reductase. Eur J Biochem.

[CR4] Bellin D, Peressotti E, Merdinoglu D, Wiedemann-Merdinoglu S, Adam-Blondon A-F, Cipriani G, Morgante M, Testolin R, Di Gaspero G (2009). Resistance to *Plasmopara viticola* in grapevine ‘Bianca’ is controlled by a major dominant gene causing localised necrosis at the infection site. Theor Appl Genet.

[CR5] Blasi P, Blanc S, Wiedemann-Merdinoglu S, Prado E, Rühl EH, Mestre P, Merdinoglu D (2011). Construction of a reference linkage map of *Vitis amurensis* and genetic mapping of *Rpv8*, a locus conferring resistance to grapevine downy mildew. Theor Appl Genet.

[CR6] Bradski G (2000). The OpenCV library. Dr Dobbs J.

[CR7] Broman KW, Sen Ś (2009). A guide to QTL mapping with R/qtl.

[CR8] Broman KW, Wu H, Sen Ś, Churchill GA (2003). R/qtl: QTL mapping in experimental crosses. Bioinformatics.

[CR9] Buonassisi D, Colombo M, Migliaro D, Dolzani C, Peressotti E, Mizzotti C, Velasco R, Masiero S, Perazzolli M, Vezzulli S (2017). Breeding for grapevine downy mildew resistance: a review of “omics” approaches. Euphytica.

[CR10] Cadle-Davidson L (2008). Variation within and between *Vitis* spp. for foliar resistance to the downy mildew pathogen *Plasmopara viticola*. Plant Dis.

[CR11] Collins NC, Thordal-Christensen H, Lipka V, Bau S, Kombrink E, Qiu J-L, Huckelhoven R, Stein M, Freialdenhoven A, Somerville SC, Schulze-Lefert P (2003). SNARE-protein-mediated disease resistance at the plant cell wall. Nature.

[CR12] Divilov K, Wiesner-Hanks T, Barba P, Cadle-Davidson L, Reisch BI (2017). Computer vision for high-throughput quantitative phenotyping: a case study of grapevine downy mildew sporulation and leaf trichomes. Phytopathology.

[CR13] Endelman JB (2011). Ridge regression and other kernels for genomic selection with R package rrBLUP. Plant Genome.

[CR14] Frazee AC, Pertea G, Jaffe AE, Langmead B, Salzberg SL, Leek JT (2015). Ballgown bridges the gap between transcriptome assembly and expression analysis. Nat Biotech.

[CR15] Hyma KE, Barba P, Wang M, Londo JP, Acharya CB, Mitchell SE, Sun Q, Reisch B, Cadle-Davidson L (2015). Heterozygous mapping strategy (HetMappS) for high resolution genotyping-by-sequencing markers: a case study in grapevine. PLoS ONE.

[CR16] Jaillon O, Aury J-M, Noel B, Policriti A, Clepet C, Casagrande A, Choisne N, Aubourg S, Vitulo N, Jubin C, Vezzi A, Legeai F, Hugueney P, Dasilva C, Horner D, Mica E, Jublot D, Poulain J, Bruyère C, Billault A, Segurens B, Gouyvenoux M, Ugarte E, Cattonaro F, Anthouard V, Vico V, Fabbro CD, Alaux M, Gaspero GD, Dumas V, Felice N, Paillard S, Juman I, Moroldo M, Scalabrin S, Canaguier A, Clainche IL, Malacrida G, Durand E, Pesole G, Laucou V, Chatelet P, Merdinoglu D, Delledonne M, Pezzotti M, Lecharny A, Scarpelli C, Artiguenave F, Pè ME, Valle G, Morgante M, Caboche M, Adam-Blondon A-F, Weissenbach J, Quétier F, Wincker P (2007). The grapevine genome sequence suggests ancestral hexaploidization in major angiosperm phyla. Nature.

[CR40] Kang HM, Zaitlen NA, Wade CM, Kirby A, Heckerman D, Daly MJ, Eskin E (2008). Efficient control of population structure in model organism association mapping. Genetics.

[CR17] Kim D, Langmead B, Salzberg SL (2015). HISAT: a fast spliced aligner with low memory requirements. Nat Method.

[CR41] Kono A, Sato A, Reisch B, Cadle-Davidson L (2015). Effect of detergent on the quantification of grapevine downy mildew sporangia from leaf discs. HortScience.

[CR18] Kortekamp A, Zyprian E (1999). Leaf hairs as a basic protective barrier against downy mildew of grape. J Phytopathol.

[CR19] Kortekamp A, Wind R, Zyprian E (1998). Investigation of the interaction of *Plasmopara viticola* with susceptible and resistant grapevine cultivars. J Plant Dis Prot.

[CR20] Luttinen J (2013) BayesNet. https://github.com/jluttine/tikz-bayesnet

[CR21] Ma Z-Y, Wen J, Ickert-Bond SM, Chen L-Q, Liu X-Q (2016). Morphology, structure, and ontogeny of trichomes of the grape genus (*Vitis*, Vitaceae). Front Plant Sci.

[CR22] McCulloch CE, Searle SR (2001). Generalized, Linear, and Mixed Models.

[CR23] McDonald BA, Linde C (2002). Pathogen population genetics, evolutionary potential, and durable resistance. Annu Rev Phytopathol.

[CR24] Moreira FM, Madini A, Marino R, Zulini L, Stefanini M, Velasco R, Kozma P, Grando MS (2011). Genetic linkage maps of two interspecific grape crosses (*Vitis* spp.) used to localize quantitative trait loci for downy mildew resistance. Tree Genet Genomes.

[CR25] Pearl J (1988). Probabilistic reasoning in intelligent systems: networks of plausible inference.

[CR26] Pertea M, Pertea GM, Antonescu CM, Chang T-C, Mendell JT, Salzberg SL (2015). StringTie enables improved reconstruction of a transcriptome from RNA-seq reads. Nat Biotech.

[CR27] Poland JA, Balint-Kurti PJ, Wisser RJ, Pratt RC, Nelson RJ (2009). Shades of gray: the world of quantitative disease resistance. Trends Plant Sci.

[CR28] Reisch B, Robinson WB, Kimball K, Pool R, Watson J (1983). ‘Horizon’ Grape. HortScience.

[CR29] Scutari M (2010). Learning Bayesian networks with the bnlearn R Package. J Stat Softw.

[CR30] Scutari M, Nagarajan R (2013). Identifying significant edges in graphical models of molecular networks. Artif Intell Med.

[CR31] Scutari M, Howell P, Balding DJ, Mackay I (2014). Multiple quantitative trait analysis using Bayesian networks. Genetics.

[CR32] The UniProt Consortium (2017). UniProt: the universal protein knowledgebase. Nucleic Acids Res.

[CR33] URGI (2014) 12X.2 version of the grapevine reference genome sequence from The French-Italian Public Consortium (PN40024). https://urgi.versailles.inra.fr/Species/Vitis/Data-Sequences/Genome-sequences

[CR34] Venuti S, Copetti D, Foria S, Falginella L, Hoffmann S, Bellin D, Cindrić P, Kozma P, Scalabrin S, Morgante M, Testolin R, Di Gaspero G (2013). Historical introgression of the downy mildew resistance gene *Rpv12* from the Asian species *Vitis amurensis* into grapevine varieties. PLoS ONE.

[CR35] Vitulo N, Forcato C, Carpinelli EC, Telatin A, Campagna D, D’Angelo M, Zimbello R, Corso M, Vannozzi A, Bonghi C, Lucchin M, Valle G (2014). A deep survey of alternative splicing in grape reveals changes in the splicing machinery related to tissue, stress condition and genotype. BMC Plant Biol.

[CR36] VIVC (2017) Table of loci for traits in grapevine relevant for breeding and genetics. http://www.vivc.de/index.php?r=dbsearch%2Fdataonbreeding

[CR37] Wang Z, Gerstein M, Snyder M (2009). RNA-Seq: a revolutionary tool for transcriptomics. Nat Rev Genet.

[CR38] Wingard SA (1941). The nature of disease resistance in plants. I. Bot Rev.

[CR39] Zhong S, Joung J-G, Zheng Y, Y-r Chen, Liu B, Shao Y, Xiang JZ, Fei Z, Giovannoni JJ (2011). High-throughput Illumina strand-specific RNA sequencing library preparation. Cold Spring Harb Protoc.

