# Supplementary material for: Single and multiple phenotype QTL analyses of downy mildew resistance in interspecific grapevines
Source: Theor Appl Genet. 2018 Feb 7;131(5):1133–43. doi: 10.1007/s00122-018-3065-y (PMC5895686; doi:10.1007/s00122-018-3065-y)
Supplement: Supplementary file 1 — Electronic supplementary material 1 (PDF 123 kb) [file 122_2018_3065_MOESM1_ESM.pdf]

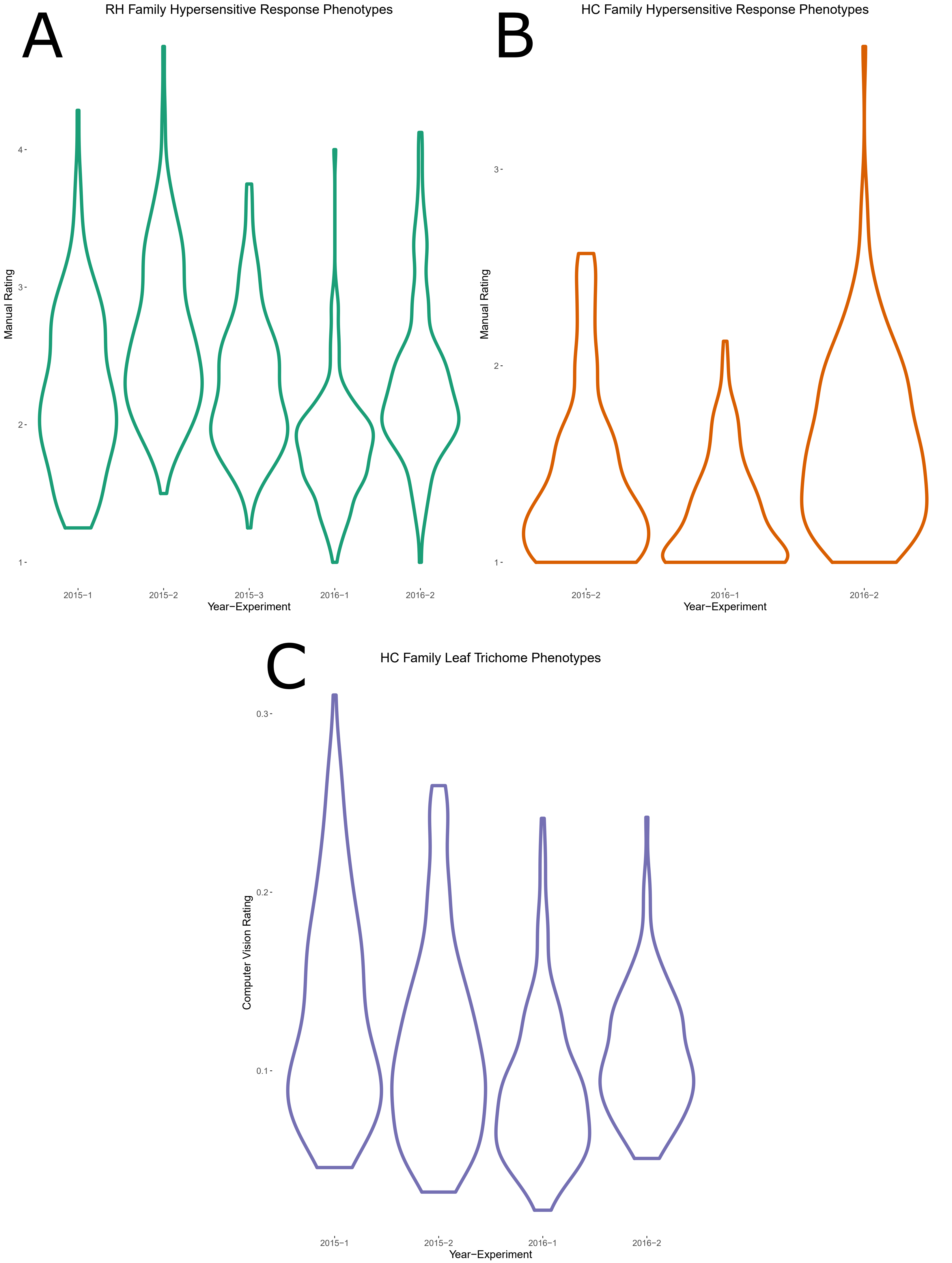

Supplementary Figure 1. Violin plots showing the **A** *Vitis rupestris* B38 x Horizon (RH) and **B** Horizon x *V. cinerea* B9 (HC) hypersensitive response (HR) phenotype distributions and the **C** HC leaf trichome phenotype distribution across experiments within years. Each phenotype is represented as the average of eight leaf disc ratings.
